# An experimental study on the effects of a simulation game on students’ clinical cognitive skills and motivation

**DOI:** 10.1007/s10459-015-9641-x

**Published:** 2015-10-03

**Authors:** Mary E. W. Dankbaar, Jelmer Alsma, Els E. H. Jansen, Jeroen J. G. van Merrienboer, Jan L. C. M. van Saase, Stephanie C. E. Schuit

**Affiliations:** Institute of Medical Education Research, Erasmus MC, University Medical Center Rotterdam, Room Ae-234, PO Box 2040, 3000 CA Rotterdam, The Netherlands; Department of Internal Medicine, Erasmus MC, University Medical Center Rotterdam, Rotterdam, The Netherlands; Department of Emergency Medicine, Erasmus MC, University Medical Center Rotterdam, Rotterdam, The Netherlands; School of Health Professions Education, Maastricht University, Maastricht, The Netherlands

**Keywords:** Simulation game, Fidelity, Cognitive skills, Motivation, Cognitive load

## Abstract

Simulation games are becoming increasingly popular in education, but more insight in their critical design features is needed. This study investigated the effects of fidelity of open patient cases in adjunct to an instructional e-module on students’ cognitive skills and motivation. We set up a three-group randomized post-test-only design: a control group working on an e-module; a cases group, combining the e-module with low-fidelity text-based patient cases, and a game group, combining the e-module with a high-fidelity simulation game with the same cases. Participants completed questionnaires on cognitive load and motivation. After a 4-week study period, blinded assessors rated students’ cognitive emergency care skills in two mannequin-based scenarios. In total 61 students participated and were assessed; 16 control group students, 20 cases students and 25 game students. Learning time was 2 h longer for the cases and game groups than for the control group. Acquired cognitive skills did not differ between groups. The game group experienced higher intrinsic and germane cognitive load than the cases group (*p* = 0.03 and 0.01) and felt more engaged (*p* < 0.001). Students did not profit from working on open cases (in adjunct to an e-module), which nonetheless challenged them to study longer. The e-module appeared to be very effective, while the high-fidelity game, although engaging, probably distracted students and impeded learning. Medical educators designing motivating and effective skills training for novices should align case complexity and fidelity with students’ proficiency level. The relation between case-fidelity, motivation and skills development is an important field for further study.

**Electronic supplementary material:**

The online version of this article (doi:10.1007/s10459-015-9641-x) contains supplementary material, which is available to authorized users.

## Introduction

Although simulation training is becoming widely established within medical education, too little attention is often paid to its effects on motivation and the clinical context (Kneebone 2005). Technology-enhanced simulation training includes computer based simulators, high-fidelity and static mannequins and training with animals or cadavers (Cook et al. 2011). It provides learning opportunities for controlled skills practice, without harming the patient. Simulation games can offer learning tasks and instruction in a realistic, engaging computer-based environment, in which trainees directly experience the consequences of their decisions (Sitzmann 2011). Well-designed simulation games (or digital games in general) apply game characteristics (players compete in attaining game objectives by following rules and principles) to create a challenging, immersive learning environment which may lead the player into a flow experience (Huang et al. 2010; Kiili 2005). Tasks in medical simulation games may contain more or less realistic cases, which can be performed repeatedly without extra costs. Games and simulations are part of the shift from a traditional training model to a learner-centred model, putting the learner into a more active role with challenging and engaging forms of learning (Garris et al. 2002).

In the next few years, higher education is expected to see an increasing use of games (Johnson et al. 2013; Prensky 2001). Although several studies have been performed in the last decade, reviews on the effects of simulation or serious games on learning outcomes and motivation have shown mixed and ambiguous results (Akl et al. 2010; Connolly et al. 2012; Graafland et al. 2012; Sitzmann 2011; Wouters and van Oostendorp 2013; Young et al. 2012). Furthermore, there is little consensus on the critical design features that support learning or motivation in games (Garris et al. 2002).

In contrast, the effectiveness of technology-enhanced simulation training, in comparison with no intervention, has been well established for knowledge and skills (Cook et al. 2011; Issenberg et al. 2005). In addition, for skills outcomes different design features have proven to be effective in simulations, such as cases with a range of task difficulty, repetitive practice, interactivity and clinical variation (Cook et al. 2013; Issenberg et al. 2005). Realistic cases in simulation training have often shown to facilitate transfer to practice (Bransford et al. 2000; Schuwirth and van der Vleuten 2003). Similarly, higher-fidelity simulations in emergency care provide greater benefit than lower-fidelity simulation, although definitions of fidelity tend to vary (Ilgen et al. 2013). However, four of five studies comparing high and low-fidelity conditions in training complex skills showed no significant differences in learning outcomes; motivational outcomes were not measured (Norman et al. 2012). Recent studies suggest that fidelity is an important factor in simulation-based training, but it is multi-factorial, and the degree of realism required of a simulation is a function of the learning task, the learners and the context (Hamstra et al. 2014).

An important consideration in instructional design is cognitive load. For novice learners, high-fidelity cases may be detrimental because they provide too many ‘seductive details’ and can easily cause cognitive overload. In combination with a complex task, too much (possibly relevant) details in a high-fidelity learning environment may cause overload and impede learning. When confronted with real or high-fidelity tasks, these tasks should initially be relatively simple, decreasing intrinsic load (Van Merrienboer and Kirschner 2012). A simpler presentation of cases (e.g. paper-based) may be just as motivating and also be more profitable for students with little experience. ‘Psychological fidelity’ pertains to the degree to which a simulated task replicates the skills and psychological factors (stress, involvement) experienced in the real environment. ‘Functional fidelity’ pertains to the degree to which a simulated task environment reacts to the tasks executed by the learner in a similar way as the real task environment. Depending on the expertise of the learner, the properties of the simulation should be functionally aligned with the criterion task (Teteris et al. 2012). ‘Physical fidelity’ pertains to the degree to which the simulated task environment looks, sounds and feels like the real environment (Van Merrienboer and Kirschner 2012). Cognitive load theory assumes that working memory load is affected by intrinsic load (the intrinsic complexity of the learning tasks), extraneous load (the manner in which the tasks are presented) and germane load (the cognitive involvement or learning that actually occurs; Paas et al. 2003). With expertise development, schemes are created which organise knowledge but also reduce working memory load. Proper measurement of the different types of cognitive load can help understand why effectiveness may differ as a function of instructional formats and learner characteristics and how they may interact (Leppink et al. 2013). Reviews of cognitive load theory underline the need to find load-reducing approaches for knowledge-intensive procedures. An important theme is finding the right balance between stimulating germane processes while offering sufficient structure to avoid cognitive overload (de Jong 2010). The role of motivation has increasingly been recognized and integrated in cognitive load theory (Paas et al. 2005).

Training emergency skills is critical for patient safety and substantial resources are involved in training this clinical cognitive skill. It involves systematic evaluation and stabilization of critically ill patients, using the internationally standardized ABCDE approach (Airway, Breathing, Circulation, Disability, Exposure). This method includes several sub skills (procedures) in which the initial resuscitation of critically ill patients is prioritized. We developed a simulation game for residents, where this cognitive skill is trained in a realistic emergency setting, as a preparation for face-to-face training. Our previous study on the effectiveness of this game showed positive effects on residents’ clinical cognitive skills (Dankbaar et al. 2014a). However, this study was inconclusive on answering the question which design features of the simulation game had been responsible for the positive effect.

In the present study, we therefore investigate the effects of adding low-fidelity and high-fidelity patient cases to an instructional e-module on medical students’ cognitive skills and motivation to learn with these instructional formats. In this experimental study we used the same simulation game as in our previous study (a virtual emergency care department) as a high-fidelity learning environment. As a low-fidelity environment, we offered the same patient cases as online text-based interactive cases. Both groups started with an instructional e-module on emergency care. We also added a control group, who exclusively studied this e-module. We chose pre-clerkship (4th-year) medical students as the target population, as they have basic knowledge of emergency care, whereas residents have typically been exposed to these skills before or during their residency program.

We hypothesized that (1) the game group would outperform the cases group on emergency skills, and that the cases group would perform better than the control group on these skills. We expected working on open patient cases would improve performance (compared to instruction only) and we expected the game would stimulate engagement and performance. (2) We expected the high-fidelity game group to be more motivated, study longer, and be more cognitively involved than the low-fidelity cases group.

## Methods

### Setting, selection of participants and design

The study was conducted at the Erasmus MC Medical School, Rotterdam, the Netherlands, with medical students who had finished their Bachelor degree, usually in their 4th-year of study, just before their 2-year clinical clerkships. The basics of emergency medicine are taught in the bachelor curriculum, but this does not include clinical training with simulation patients. Emergency care skills are clinical, primarily cognitive skills: the ability to perform primary assessment of a seriously injured or ill patient, to determine the priority of the necessary actions, and to start initial treatment (Breedveld 2009).

The recruitment took place in March 2014 after a 4th-year lecture and through the Medical School’s online communication channel for 4th-year medical students. Students were recruited 5 weeks before the assessment to allow time for self-study at home. When they consented to participate they were randomly assigned (using the Excel random function) to the game, cases or control group and received a personal access code. An i-Pad was raffled among the participants who showed up at the assessment. The skill assessments were carried out from 14 to 18 April 2014.

#### Study design

**Fig. 1:**
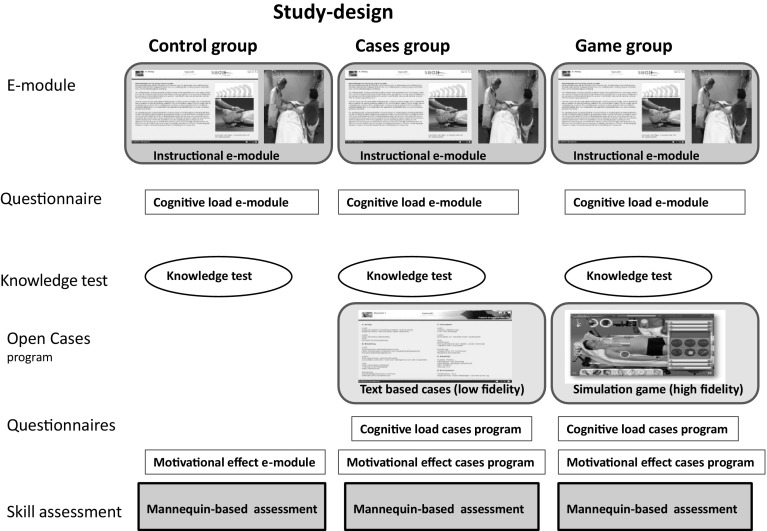
Study-design

The control group had access from home to an instructional e-module on emergency care, followed by a knowledge test. The cases group, in addition to the e-module, worked on online text-based cases (‘low-fidelity condition’). The game group, in addition to the e-module, worked on a simulation game with the same cases (‘high-fidelity condition’). All groups completed questionnaires on motivation and cognitive load after working on the study material. After 4 weeks of study-time, participants did a mannequin-based scenario assessment. As inter-case variability is known to be an important source of error (Norman 2002; Swanson and van der Vleuten 2013) all students did an assessment with two cases (Fig. 1).

### Materials

#### E-module

All groups started their online training with an instructional e-module on emergency care, aimed at developing knowledge on systematically evaluating and stabilizing acutely ill patients, using the internationally standardized ABCDE approach (Airway, Breathing, Circulation, Disability, Exposure). In this approach the Airway, Breathing and Circulation are assessed, using the “look, listen, feel” steps. The information was presented in text with photos, questions with feedback and a 15-min demonstration video (‘worked case’) by an experienced doctor and nurse with a standardized patient. The e-module contained no open cases and took about 2 h to study; students were free to return to the e-module during their work on the cases.

#### Knowledge test

The self-diagnostic knowledge test consisted of 24, four options Multiple Choice (MC) questions on emergency care. After finishing the test, a fail/pass result was presented (the standard was 75 % correct) with the number of correct answers. Students could do the test up to 3 times; the last score was saved.

#### Text-based cases

The text-based cases program was developed for the research study and was designed to train the ABCDE approach. It offered six online, interactive text-based patient cases, each with one patient photo with brief information on the patients’ condition and open questions on the initial assessment and treatment of the patient, followed by written feedback. For instance: ‘where do you start with this patient and where do you focus on in the physical examination?’ was followed by a chronological description of the assessment of the patient (learners can compare their answers). All information on the patients’ condition was provided in text; the patient did not react to wrong decisions or interventions. The six patient cases were about acutely ill patients, e.g. gastro-intestinal bleeding leading to circulatory shock and subarachnoid bleeding with seizures. If a case was done for a second time, the same questions and feedback were provided. This *low*-*fidelity* condition provided lower functional fidelity (feedback on the actions of the learner was text-based, thus less similar to the real task) and lower physical fidelity (the task environment looked and sounded less like a real clinical environment), compared to the game condition. We estimated the cases program took 2–4 h to study (screenshots of the Cases are in supplementary file 1).

#### Simulation game (‘abcde*SIM*’)

The computer-based simulation game (abcde*SIM*) was designed for residents to train cognitive emergency care skills (perform the ABCDE approach on different patients). Its design was based on analysing the task demands of this approach and offered an online realistic emergency department with the same six cases, which now took the form of virtual patients. The game started with a storyline in which the patient was presented to the player with brief information on the patients’ condition. The player then performed physical examination, aided with tools (e.g. a penlight symbolizes “look”, a stethoscope symbolizes “listen” and a hand symbolizes “feel”) which had to be used in a correct way to gather the correct information. The stethoscope would only provide breathing sounds if put on the right spot on the thorax, and different locations provided different lung sounds. The player could also order diagnostics (e.g. laboratory testing, ECG); results were presented after a certain timeframe. All sounds, pupil reflexes, lab results etc. had to be acquired and interpreted by the player and this information should lead to proper treatment of the patient with oxygen, infusion fluids, medication, etc. The (absence of) treatment lead to changes in the patients’ vital signs, real time displayed on a medical monitor (with sounds) to increase fidelity and give immediate feedback on the patients’ condition. The vital signs were generated by a high-fidelity model of human physiology. Each patient had to be stabilized within 15 min; a timer was presented. The storyline ends with an explanation of how the patient faired after the players care, including narrative feedback on the actions and a score. The score depended on the number of correct decisions taken according to the ABCDE approach (e.g. put right oxygen mask on patient: 20 points) and the efficiency of actions (less minutes means more points). Each time a game-case was played, the condition of the patient could vary, depending on the interventions made. Players could compare their scores with peers and with a ‘high score’ list, to stimulate competition between players. The game did not explicitly train communication skills. This *high*-*fidelity* condition provided higher functional fidelity (alignment of cognitive demands in the game tasks with the real tasks, including realistic feedback from the patient’s reaction on the learner’s actions) and higher physical fidelity (the sub tasks, such as using the stethoscope and tools, such as a vital sign monitor looked and sounded like the ones in a real emergency department). The psychological fidelity, related to the cognitive skill at stake including stress, boredom etc. was similar in both conditions. We estimated the game took 2–4 h to study (screen shots of the Game are in supplementary file 2).

#### Skill assessment

Students were assessed at our institution with two 15-min mannequin-based (Laerdal Crash Kelly^®^) scenario assessments on diabetic coma and urosepsis with delirium (in random sequence), taking their low level of experience into account. In both scenario’s, students had to work according to the ABCDE-approach, as taught in the E-module. Assessors, who were qualified instructors in emergency courses, introduced and led the scenario. They were familiar with the assessment instrument, blinded for the intervention group and changed scenarios halfway. To get a passing score for the scenario, student had to make a proper diagnosis and start treatment, showing adequacy in the skills that were assessed. The assessment instrument consisted of a *clinical competency scale* (6 items on the ABCDE method), a *communication competency scale* (3 items on communication with the patient and nurse), both rated on a 7-point scale (1 = very weak, 7 = excellent), and a *global performance scale* (10-point scale to rate ‘independent functioning in caring for acutely ill patients in the Emergency Department’, 10 = perfect). The construct validity and inter-rater reliability of the assessment instrument was validated in a separate study with residents. The clinical competency scale had good construct validity (factor-analysis explained 65 % of total variance); the clinical competency and global performance scale had moderate interrater reliability (ICC single measures = 0.49/0.50^1^); the communication competency scale had poor interrater reliability (ICC = 0.27). Internal consistency of scales was high (α = 0.91 and 0.86 for clinical and communication competency scales resp.; Dankbaar et al. 2014b).

#### Questionnaire on cognitive load

The cognitive load questionnaire has been validated in other work (Paas et al. 2003). It consisted of 10 questions; 3 on *intrinsic cognitive load* (e.g. “the content of the e-module/cases/game was very complex”), 3 on *extraneous cognitive load* (e.g. “the explanations and instructions in the e-module/cases/game were very unclear”) and 4 on *germane cognitive load* (e.g. “the e-module/cases/game really enhanced my understanding of the problems that were discussed”). The questionnaire was scored from 0 (= not all applicable) to 10 (= very much applicable). Sores ≥ 6 for intrinsic load indicate that content is perceived as complex.

#### Questionnaire on motivation and learning time

A questionnaire on motivation to learn with one of the three formats was developed, based on a previous study on the game effectiveness with residents (Dankbaar et al. 2014a). The 9-item questionnaire consisted of 2 constructs: engagement (“it was fun to work through the material”, 6 items) and feedback (“during learning, I could tell whether I was doing well”, 3 items); scored on a 5-point Likert scale (1 = fully disagree, 5 = fully agree). For the control group, the item on the challenging character of cases was left out, leaving 8 items for this group (questionnaire can be found as supplementary file 3). At the end of this questionnaire, participants also answered some general questions on age, gender, the amount of time they spent on the study material, their clinical experience and whether they had experience with acutely ill patients. All participants reported learning time separately for the e-module and (if applicable) for the cases or the game. Game-time was also computer-recorded.

### Statistical analysis

We assessed associations between categorical variables using Chi squared tests. ANOVA analysis and independent *t* tests were used to compare groups on continuous and ordinal variables. Unless the distribution of scores is severely skewed, data from rating scales can be analyzed as if they were interval scales without introducing bias (Streiner and Norman 2008). For statistically significant findings in ANOVA, post hoc analysis was done with S–N–K adjustments. Effect sizes (ES) were calculated using Glass’s delta, as homoscedasticity (same variance) is not assumed (Grissom and Kim 2012). Effect sizes of 0.50 were considered medium and ES ≥0.80 were considered large (Hojat and Xu 2004). We used Pearson’s coefficient to calculate associations. Inter-case reliability in performance assessment was calculated using the Intraclass correlation (ICC). We used regression analysis to determine the factors that were related to clinical skill levels. Analyses were performed using SPSS; we present 95 % confidence intervals (CIs).

#### Ethical issues

The study was approved by the Dutch ethical board for research in Medical Education (NVMO, no. 210). All participants signed an informed consent form.

## Results

### Student characteristics

In total 79 students were recruited for the study; they were randomly assigned to the game (n = 30), cases (n = 30) and control group (n = 19). As we were particularly interested in the effects of fidelity on motivation and performance in the cases and game group, we chose to make the control group smaller (25 % of the participants). In total 61 students showed up for the assessment 4 weeks later (77 %); 25 from the game, 20 from the cases and 16 from the control group. There were no significant differences between groups in ‘no shows’. The participating students on average were 23 years old, had some clinical experience (in clerkship or with acute patients); a small majority was female. There were no differences between intervention groups in age, clinical experience, or grade point average (GPA) in the Bachelor, but groups did differ in gender: overall 48 % of the participants were male, but the control group had more male students (69 %) than the game group (28 %; χ^2^ (2) = 7.2, *p* = 0.03).

### Knowledge test

There were no significant differences in results on the knowledge test between the three intervention groups. The game group had a score of 20.4 (SD = 2.1), the cases group 19.7 (SD = 3.4) and the control group 19.4 (SD = 5.3) out of 24 points. Thus, all groups had the same knowledge score after doing the e-module (mean difference between control and cases group: −0.12; 95 % CI −3.23 to 2.99; between control and game group: −0.64; CI −3.57 to 2.30; between cases and game group: −0.52; CI −3.30 to 2.27, *p* = 0.84).

### Assessment results emergency care skills

There were no significant differences between groups in assessment scores for clinical competencies (M = 5.4), communication competencies (M = 5.2), and global performance (M = 7.5), Table 1). Mean differences with confidence intervals are presented in Table 2. There was no difference in skills between male and female students. The internal consistency (α) of the competency scales for case 1 was 0.84 and 0.86 for the clinical and communication scale respectively; for case 2 it was 0.83 and 0.81.

**Table 1:** Assessment scores per intervention group (average scores over 2 scenarios)

	Total n = 61	Control group n = 16	Cases group n = 20	Game group n = 25	F	*p* value
	Mean (SD)	Mean (SD)	Mean (SD)	Mean (SD)		
Clinical competencies (6 items, 7-point scale)	5.4 (0.71)	5.6 (0.64)	5.2 (0.75)	5.4 (0.68)	2.10	0.13
Communication competencies (3 items, 7-point scale)	5.5 (0.52)	5.7 (0.35)	5.4 (0.59)	5.5 (0.52)	2.69	0.08
Global performance (10-point scale)	7.5 (1.0)	7.9 (0.89)	7.2 (1.1)	7.4 (1.0)	2.22	0.12

**Table 2:** Mean differences and confidence intervals for assessment scores

	Mean difference	95 % CI
*Clinical competencies*		
Control—cases group	0.48	−0.10 to 1.10
Control—game group	0.25	−0.30 to 0.80
Cases—game group	−0.23	−0.74 to 0.29
*Communication competencies*		
Control—cases group	0.38	−0.04 to 0.80
Control—game group	0.29	−0.11 to 0.69
Cases—game group	−0.10	−0.47 to 0.28
*Global performance*		
Control—cases group	0.71	−0.13 to 1.55
Control—game group	0.46	−0.35 to 1.26
Cases—game group	−0.26	−1.01 to 0.49

The inter-case reliability as measured with ICC (single cases) was 0.54 for global performance, 0.41 for the communication competencies and 0.39 for the clinical competencies.

### Learning time and game data

Self-reported total learning time was higher for both the game and cases groups (ca. 4 h) in comparison with controls (ca. 2 h), with large variation within groups [F(2, 56) = 5.1, *p* ≤ 0.05, Table 3]. All groups spent the majority of their learning time on the e-module. The cases and game group did not only spend additional time on the cases and game (70 resp. 95 min), but also spent more time on the e-module compared to the controls, although this difference was not statistically significant [F(2,56) = 0.96, *p* > 0.10]. There was no significant difference in total learning time between the cases group and the game group (mean difference −6 min; 95 % CI −96 to 84, *p* = 1.0). Two students in the game group did not use the game (they were included because there was an ‘intention to treat’).

**Table 3:** Self-reported learning time per intervention group (in min)

	Control group n = 16	Cases group n = 20	Game group n = 25	F	*p* value
	Mean (SD)	Mean (SD)	Mean (SD)		
E-module	133 (78)	177 (118)	146 (98)	0.96	0.39
Game/cases		70 (46)	95 (53)	2.7	0.11
Total self-reported learning time	133 (78)	247 (139)	241 (124)	5.1	0.01*
Logged game time (min)			90 (49)		

* Significantly different between control and cases/game group

Analysis of performance of longer playing students in the game group (above the median of 88 min, n = 12), compared to shorter playing students showed slightly higher scores for clinical competencies (M = 5.6) and global performance (M = 7.8), but they did not outperform the control group. Communication competencies remained the same (M = 5.5).

The mean game score was 3172 points (SD = 1599). There was an association between game time and game score (r = 0.80, *p* < 0.0001), and between game time and clinical competencies (r = 0.45, *p* = 0.03). There was no association between self-reported time (on text-based or game-based cases) and clinical competencies (*r* = −0.12).

#### Factors influencing skills level

In order to analyse which factors determine the level of clinical competencies, a regression analysis was performed with the variables: intervention group, experience (in clerkship or with acute patients), grade point average (GPA), and total self-reported learning time. GPA was the only significant factor predicting skill level in the total group of students (B = 1.89, *p* = 0.01). No association between GPA and game time existed (r = 0.05, *p* > 0.1), indicating independence of effects.

### Cognitive load

**Fig. 2:**
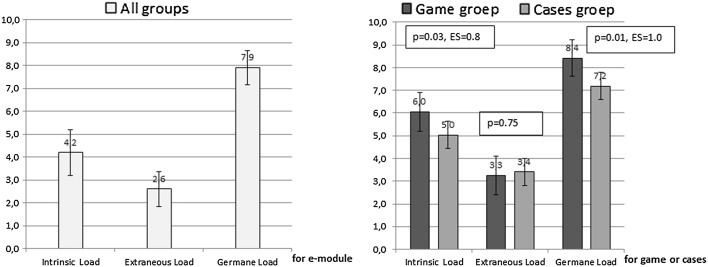
Cognitive load scores for the e-module (all groups) and for the game and cases

The mean intrinsic cognitive load score of the e-module for all groups was 4.2 (SD = 2.02, n = 54), extraneous cognitive load was 2.6 (SD = 1.52) and germane cognitive load was 7.9 (SD = 1.50). Comparing cognitive load of the e-module with cognitive load of the cases or game (for the cases/game group, using a paired *t* test) showed that only *intrinsic* cognitive load of the e-module (M = 4.7, SD = 2.1) was lower than intrinsic load of the cases or game (M = 5.7, SD = 1.6, *p* = 0.02, ES = 0.45). The game group experienced significantly higher levels of intrinsic and germane cognitive load than the cases group with large effect sizes, but comparable extraneous cognitive load (Fig. 2).

In summary, the e-module content was not perceived as very complex, the explanations were clear and students were very actively processing the information from the e-module. The game was perceived as more complex than the cases program and students were more actively processing the tasks. Explanations in the game and cases program were equally clear.

### Motivation questionnaire

**Fig. 3:**
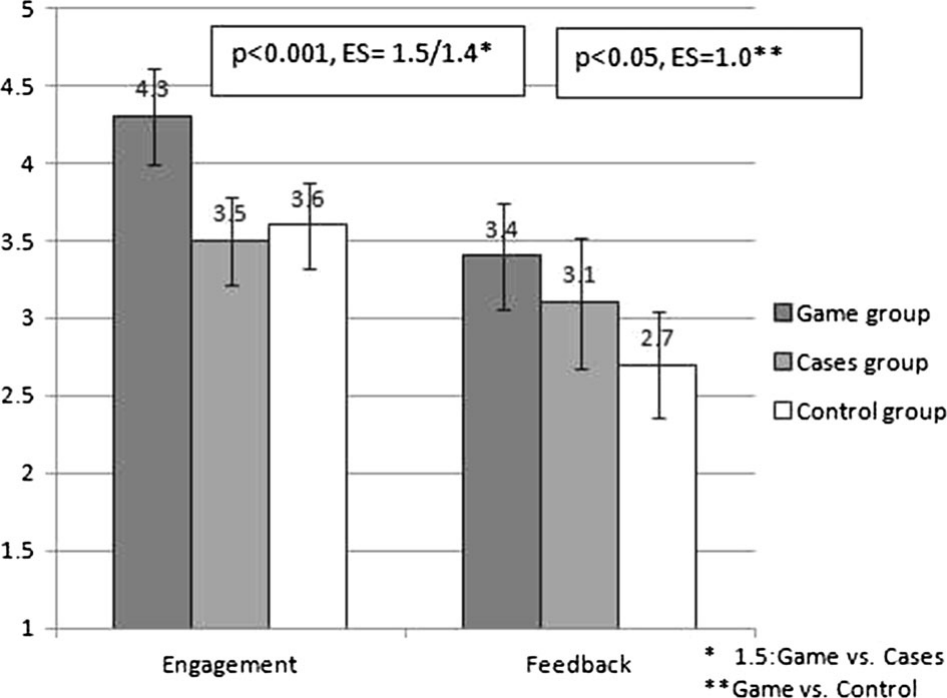
Engagement and feedback scores per intervention group for the e-module, cases and game

Mean scores on engagement were different for the three groups (Fig. 3). The game group experienced higher engagement than both the cases and control groups (both *p* < 0.001), with large effect sizes. The game group experienced more feedback than the control group (*p* < 0.05, with large effect sizes), but not compared to the cases group (*p* = 0.2). There was no association between the engagement scores and game time (r = 0.22, *p* > 0.1).

In open remarks from students from the game group they stated they liked learning with the game, but would have preferred more specific feedback on their actions after doing a case and would have liked additional instructional demonstration videos including the use of instruments.

## Discussion

This study compared the effects of high-fidelity and low-fidelity open patient cases and a control condition (working on an e-module only) on students’ cognitive skills and motivation. We showed that complex cognitive emergency care skills of 4th-year medical students were not improved by adding open patient cases as part of a (high-fidelity) simulation game or (low-fidelity) text-based cases to an instructional e-module. Students who worked on the patient cases experienced them as more complex than the instructional e-module, and spent an additional 2 h studying. Surprisingly, despite this extra study-time, their performance in two scenario-based assessments did not improve. A second finding was that the simulation game, that considerably enhanced students’ motivation, was perceived as more complex and stimulated students to put more effort in the cases compared to the text-based cases. Nonetheless, the game group did not study longer and showed the same level of performance as the text-based cases group. The levels of extraneous load were equally low for both cases groups, indicating learning was not hampered by unclear explanations.

Our first hypothesis that the game group would outperform the cases group, and the cases group would outperform the control group on emergency skills was clearly not confirmed. All groups showed the same performance in emergency care, despite large differences in learning time. Our second expectation that the game group would be more motivated and show more cognitive involvement than the cases group was confirmed, although, despite their higher engagement, they did not study longer and did not improve their performance.

Why did these 4th-year students not profit from the 2 h of highly engaged, extra learning time spent on the text- and game-based cases? All groups showed the same knowledge levels on the self-test after studying the e-module. Maybe our study was underpowered and real, existing differences remained unnoticed? Although we cannot exclude this possibility, we believe it is unlikely this would have changed our main conclusions, considering the assessment scores and confidence intervals around the differences of the experimental groups. Apparently the e-module, including a demonstration video on the ABCDE approach, was quite effective. Hence a possible explanation is we observed a ceiling effect and the cases had nothing to add in terms of learning. Although the assessment scores may seem relatively high, assessors took the low level of students’ experience in emergency care into account; as a result these scores cannot be interpreted in absolute terms. The fact that students spent considerable extra time on the cases, and reported high levels of germane load in both conditions makes this assumption unlikely. The opposite argument, the possibility that the assessment setting with a mannequin was too difficult for the students, is in fact more likely. Working with mannequins is not implemented in our medical curriculum and is different from working with simulated cases. Maybe the assessment instrument also was not sufficiently differentiating lower skills levels? The high reliability scores of the competency scales, indicating good variability within groups, and the good inter-case reliability do not provide support for this assumption. The fact that neither the cases nor the game group improved learning indicates that students probably experienced a problem with the open patient cases; we expect they were too complex to solve without guidance. This is confirmed by the high germane load scores in both case conditions and the fact that the intrinsic load in these conditions was perceived as higher compared to the intrinsic load in the e-module. Moreover, the 2 h extra learning time was only partly spent on the cases; the majority of the 4 h learning time was spent on the e-module. Students may have returned to the e-module for supporting information to solve the case problems. Apparently for these novices in emergency care, the cognitive gap between the e-module and the six open cases was too large. This assumption is supported by remarks from students that they would have liked more video demonstrations and more specific feedback in the cases. Spending extra time on the game and text-based cases may even have resulted in a shift from being “unconsciously incompetent” to being “consciously incompetent”, making them more insecure during assessment (but possibly more eager to learn in the future).

In summary, we believe the e-module (with the video) was quite effective in teaching the principles of the ABCDE approach, the open case problems were too complex for the students and possibly the assessment situation was too demanding as well. Our finding that for initial skills acquisition, solving open cases does not necessarily add anything in terms of learning to (online) instruction with a demonstration video, is in line with research showing that studying worked examples is superior to solving training problems, attributed to the fact that examples allow for building a cognitive scheme (Leppink et al. 2014; Stark et al. 2011). In addition, the effectiveness of online instruction for learning skills is consistently confirmed in review studies (Cook et al. 2008; Sitzmann et al. 2006), as has the effectiveness of instructional videos. For instance, in a critical care situation, students who watched a video demonstration or practiced hands-on in a high-fidelity simulator generated the same responses (Baxter et al. 2012). Another study with surgical novices showed that video training in adjunct with expert instruction did not improve the development or retention of surgical skills more than video training alone (Nousiainen et al. 2008).

Contrary to our expectations, the high-fidelity game group spent the same amount of learning time as the low-fidelity cases group although, as expected, the game group was more motivated and was more cognitively involved in learning. The cases group was less engaged to learn with their format, but apparently was sufficiently motivated to learn about the ABCDE approach, resulting in the same time on task and the same skills level.

Was the game more distracting than helpful for learning? The association between game time and clinical skills indicates functional alignment between game-tasks and assessed tasks probably did not fail completely, as this association was independent from academic performance or students’ motivation. However, since longer playing students (>90 min) still did not outperform the control group, the game is probably distracting for novices, specifically in the beginning. A possible explanation is that the attributes which created higher physical fidelity in the game (the tasks and tools in the game, which looked and sounded like a real emergency room) were very engaging for students, but at the same time confused them, creating overload in combination with the task demands, and deteriorated learning. The attributes which created higher functional fidelity (alignment of cognitive task demands with real tasks, including realistic feedback from the patients’ reactions) may be only effective after players spent more time, got used to the sounds and tools, and were able to concentrate on the cognitive tasks, if at all. Further research is needed to confirm this conjecture. Measurement of cognitive load *during* game play rather than one measurement after playing different cases in the game would be useful to gain more insight in this process. In addition, the extraneous load items (currently addressing “unclear instructions”), might be extended with items addressing distracting elements in the learning task.

The *abcdeSIM* game was originally developed for residents. Compared to 2nd year residents who did show improved clinical cognitive skills after playing the same game for (on average) 130 min, these students spent limited game-time (90 min) and also had lower game-scores (Dankbaar et al. 2014a). This is an important finding, indicating an ‘expertise reversal effect’ where a rich learning environment (with tasks at a specific level of complexity) may benefit experts, but is counter-productive for novice learners (Kalyuga et al. 2003). Another important difference between residents from the previous study (Dankbaar et al. 2014a) and students in the current study is that residents were preparing for an emergency department traineeship 2 weeks later, increasing their dedication to learn this skill. Students volunteering for a research study do not have the same focus during learning.

Is it possible that other game attributes than fidelity stimulated engagement and cognitive effort? The serious game and text-based cases mainly differed in functional and physical fidelity, but the game also had a scoring system to stimulate competition and longer play. Although we cannot exclude the possibility that engagement was positively influenced by the scoring system, competition did not lead to more time on task or higher skills levels in the game group compared to the cases group. Mayer concluded from several studies there is not yet convincing evidence that competition in games stimulates learning (Mayer 2014).

In summary: the high-fidelity simulation game enhanced motivation and cognitive effort compared to the cases, but appears to be distracting and impeded learning for novices. We expect especially the physical fidelity (tools and sounds in the game) provided too many details and might have created cognitive overload. Our finding that high-fidelity presentation of cases did not improve learning for novices is in line with a number of other studies. Formats with different levels of authenticity (paper-based cases, video cases and simulated patients) did not influence students’ skills and performance on the short and longer term (Durning et al. 2012). Two studies comparing text-based and video-enhanced virtual patients showed better respectively worse reasoning outcomes for the text-only format, with small effect sizes (Cook et al. 2010). Schematic representations in (non-medical) simulation games often were more effective than realistic representations (Wouters and van Oostendorp 2013). However, Brydges et al. (2010) found that allowing students to progress in their practice on simulators of increasing fidelity improved transfer of a broad range of clinical skills. More research is needed on the interplay between complexity of cases, (functional and physical) fidelity, motivation and performance for different proficiency levels.

### Implications for medical education

This study yields a number of implications for designing engaging and effective online skills training in medical education. For initial cognitive skill acquisition, an instructional e-module with one or more demonstration videos is a powerful instructional format. Additional patient cases stimulate students to study longer and be cognitive involved, but do not necessarily result in learning when these tasks are too complex. Worked cases, part task practice, hints and gradually increasing task complexity in open cases can be used to help students build the cognitive schemes required as a preparation for doing more complex open cases (Van Merrienboer and Kirschner 2012). Although high-fidelity patient cases in a simulation game may enhance motivation and cognitive involvement, they can easily distract novice students and impede learning. In particular physical fidelity can easily create cognitive overload for novices. Learning task fidelity should only gradually increase as learners become more proficient (Leppink and van den Heuvel 2015). As the same game, originally developed for residents, resulted in improved clinical competencies with family practice residents (Dankbaar et al. 2014a), our results from the two studies are an illustration of the *expertise reversal effect*, where a rich learning environment may benefit experts, but is counter-productive for novice learners. Our finding that motivation and cognitive involvement were unrelated to learning outcomes is an important notion for game-based learning and research, as objectively measured learning outcomes are not always part of models in use. Moreover, a simulation game that motivates and stimulates learners has little practical meaning in the absence of real learning effects.

### Limitations

This study has a number of limitations. First, the sample size of the control group was limited, as a result comparisons between the control group and the game or cases group may have been underpowered. Considering the assessment scores and confidence intervals around the differences of the experimental groups, we do not expect larger samples would have changed our conclusions. However, in a follow-up study we recommend to use larger samples for the control group. A second limitation was the fact that patient cases appeared to be too complex, which made it difficult to investigate the main research question regarding the effect of fidelity in cases. Based on the combined data from the assessment, game play and questionnaires we were able to draw some conclusions, but in a future study it is advisable to test complexity of cases before the start of a research study, e.g. in a pilot. A third limitation was the problem of confounding, as the game differed in several attributes from the text-based condition, such as physical and functional fidelity and a scoring system. We argued it is unlikely the scoring system had a considerable influence on the main results, but it was not possible to separate the effects of physical and functional fidelity. Although in games different attributes often are intertwined, for design-based research it is important to develop and investigate game versions with unique attributes. A last limitation is the fact that this study is focused on cognitive emergency care skills of 4th-year medical students. As competency level of learners is an important factor in the effectiveness of instructional design, findings cannot be generalised to other groups or domains.

## Conclusions

This study compared the effects of an emergency care simulation game with a text-based cases program (with the same cases) and a control condition (working on an e-module only) on students’ clinical cognitive skills and motivation. For initial skills acquisition, an e-module with a video demonstration is a powerful instructional format. These students, who were inexperienced in emergency care, did not profit from additional work on open cases, which appeared to be too complex, but nonetheless challenged them to study longer. The high-fidelity simulation game increased complexity and did not improve their skills-level, even though students put more effort into it and felt more engaged with it than with the low-fidelity cases. Probably the game distracted students and impeded learning. In the design of motivating and effective cognitive skills training for novices, complexity and fidelity of cases should be well aligned with students’ proficiency level. More design-based research is needed on the relation between case-fidelity, motivation and skills development for novices and experts to make informed choices in this increasingly important field.

## Electronic supplementary material

Below is the link to the electronic supplementary material.

Supplementary material 1 (DOC 36 kb)

Supplementary material 2 (JPEG 108 kb)

Supplementary material 3 (JPEG 95 kb)

## Footnotes

1 For Interrater reliability, ICC values (single measures) of 0.21–0.40 are considered ‘poor’, 0.40–0.60 ‘moderate’, 0.60–0.80 ‘substantial’ and 0.80–1.00 ‘almost perfect’.
