# Supplementary material for: An experimental study on the effects of a simulation game on students’ clinical cognitive skills and motivation
Source: Adv Health Sci Educ Theory Pract. 2015 Oct 3;21:505–21. doi: 10.1007/s10459-015-9641-x (PMC4923100; doi:10.1007/s10459-015-9641-x)
Supplement: Supplementary file 1 — Supplementary material 1 (DOC 36 kb) [file 10459_2015_9641_MOESM1_ESM.doc]

**Supplementary file 3: Motivation questionnaire**

Choose the assertion which fits best your opinion on the study material

|  |  | **1 Fully disagree** | **2 Disagree** | **3 Neutral** | **4 Agree** | **5 Fully agree** |
| --- | --- | --- | --- | --- | --- | --- |
| E 1 | I think the e-module/cases/game can be valuable for me |  |  |  |  |  |
| E 2 | I think this study material is helpful for me to master the ABCDE approach |  |  |  |  |  |
| E 3 | I liked this way of learning |  |  |  |  |  |
| E 4 | It was fun to work through the material |  |  |  |  |  |
| E 5 | After working through the material, I felt encouraged to study it again |  |  |  |  |  |
| E 6 | *It was challenging to perform well on the patient cases* (not for control group) |  |  |  |  |  |
| F 1 | During learning, I could tell whether I was doing well |  |  |  |  |  |
| F 2 | I could experience for myself what did and did not work in the ABCDE approach |  |  |  |  |  |
| F 3 | I received sufficient feedback |  |  |  |  |  |

Do you have any remarks on the content of the material (positive or negative)?

Positive: ……………………………….

Negative: ……………………………..

E: Engagement Questionnaire; F: Feedback Questionnaire
