# Supplementary figures and images for: An experimental study on the effects of a simulation game on students’ clinical cognitive skills and motivation
Source: Adv Health Sci Educ Theory Pract. 2015 Oct 3;21:505–21. doi: 10.1007/s10459-015-9641-x (PMC4923100; doi:10.1007/s10459-015-9641-x)

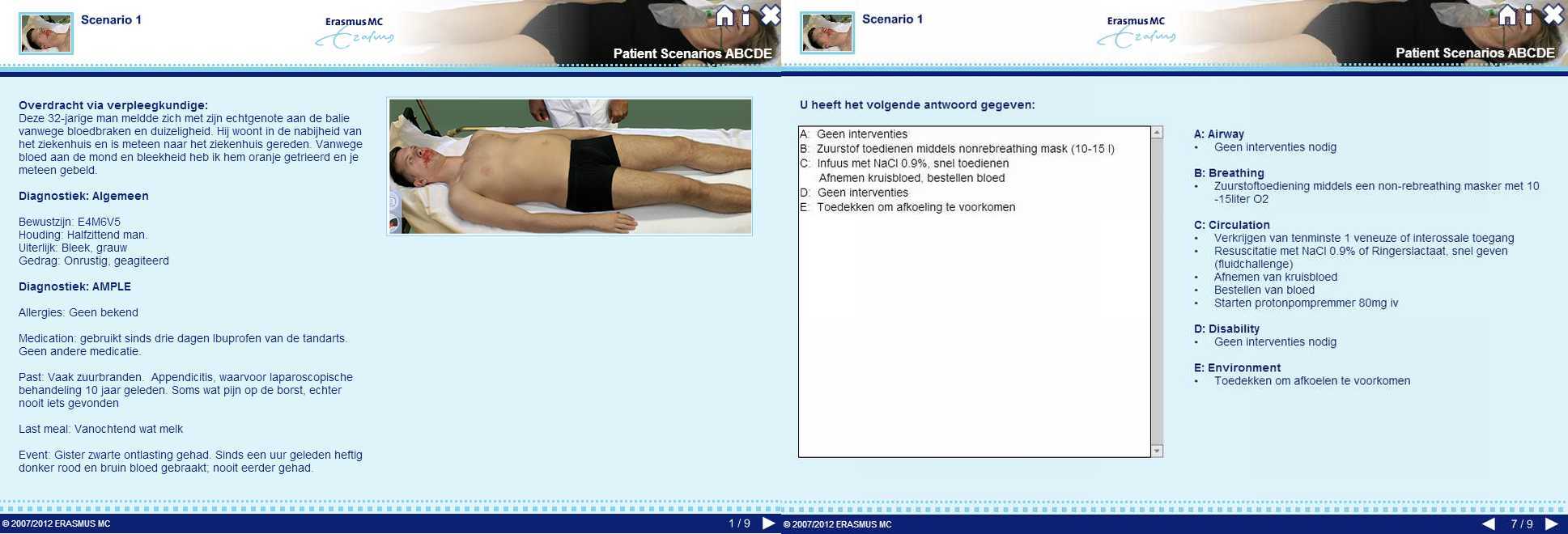

Supplement: Supplementary file 2 — Supplementary material 2 (JPEG 108 kb) [file 10459_2015_9641_MOESM2_ESM.jpg]

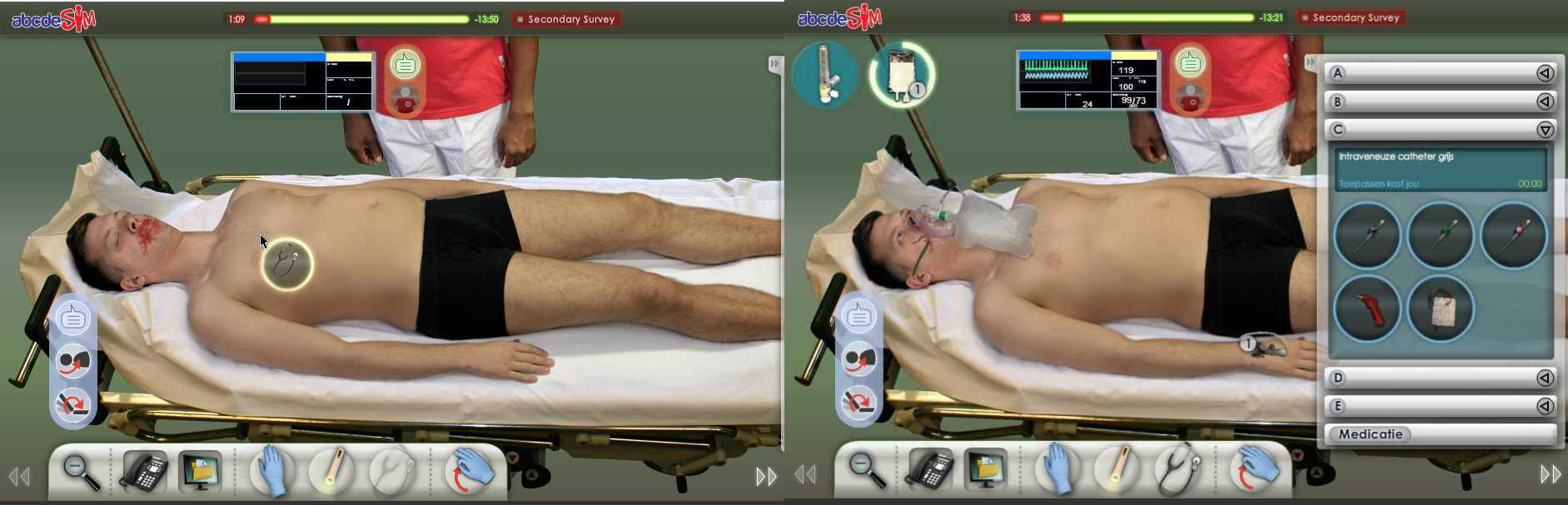

Supplement: Supplementary file 3 — Supplementary material 3 (JPEG 95 kb) [file 10459_2015_9641_MOESM3_ESM.jpg]
